# Epigenetic Regulations in Diabetic Nephropathy

**DOI:** 10.1155/2017/7805058

**Published:** 2017-03-19

**Authors:** Zeyuan Lu, Na Liu, Feng Wang

**Affiliations:** ^1^Department of Nephrology, Shanghai Jiao Tong University Affiliated Sixth People's Hospital, Shanghai, China; ^2^Department of Nephrology, Shanghai East Hospital, Tongji University School of Medicine, Shanghai, China

## Abstract

Diabetic nephropathy (DN) is a chronic complication of diabetes and the most common cause of end-stage kidney disease. It has been reported that multiple factors are involved in the pathogenesis of DN, while the molecular mechanisms that lead to DN are still not fully understood. Numerous risk factors for the development of diabetic nephropathy have been proposed, including ethnicity and inherited genetic differences. Recently, with the development of high-throughput technologies, there is emerging evidence that suggests the important role of epigenetic mechanisms in the pathogenesis of DN. Epigenetic regulations, including DNA methylation, noncoding RNAs, and histone modifications, play a pivotal role in DN pathogenesis by a second layer of gene regulation. All these findings can contribute to developing novel therapies for DN.

## 1. Introduction

Diabetic nephropathy (DN) is characterized by glomerular hypertrophy, proteinuria, decreased glomerular filtration, and renal fibrosis resulting in the loss of renal function. DN is a major complication associated with both type 1 and type 2 diabetes [1]. More than 1/2 of patients with type 2 diabetes and 1/3 of those with type 1 diabetes develop kidney disease, and DN is a prime reason for dialysis in many developed countries [2]. DN is classified as a small blood vessel complication of diabetes. Both ethnicity and inherited genetic differences are proposed for response to the development of DN [3, 4]. Intraglomerular hypertension and hyperfiltration are triggered because of the hyperglycemia, insulin resistance, and aberrant hemodynamics. However, the molecular mechanisms leading to DN are still not fully understood. Recently, there is emerging evidence that suggests the important role of epigenetic mechanisms in the pathogenesis of DN. It is rather remarkable that individuals who are exposed to hyperglycemia more likely develop diabetic complications. This phenomenon is referred to as metabolic memory or the legacy effect [5].

Epigenetics refers to heritable patterns of gene expression that are not dependent on the DNA sequence information. It has been studied in a variety of organisms [3]. The epigenetic modifications include cytosine methylation of DNA (DNA methylation), histone posttranslational modifications (PTMs), and noncoding RNAs [6]. The most well-known and best-characterized epigenetics are DNA methylation, which converts the DNA nucleotide, cytosine, into 5-methyl-cytosine. Another important epigenetic mark is the modification of histone proteins around which less than two turns of DNA are wound to form the nucleosome. Besides, noncoding RNA is regarded as an important part of epigenetic, which can regulate gene expression at both transcription and translation level [7]. Numerous studies have shown that epigenetic processes are involved in the pathogenesis of DN. In this review, we discuss recent advances in the epigenetics of DN, including DNA methylation, noncoding RNA, and histone modifications, with the focus on the role of three types of epigenetic modification in DN.

## 2. DNA Methylation in DN

DNA methylation, frequently described as a “silencing” epigenetic mark, usually occurs at 5′-cytosines of CpG dinucleotide and is catalyzed by specific DNA methyltransferases [8]. Generally, if DNA methylation exists at gene promoter regions, it will lead to gene repression. On the other hand, if DNA methylation exists at gene bodies, it can modulate transcription elongation and alternative splicing. One of the mechanisms of DNA methylation is inhibiting gene expression by methyl binding proteins, which can recruit transcriptional corepressors. Besides, another mechanism is that it can interfere with the binding of transcription factors at promoters [7].

Hyperglycemia has been reported to change DNA methylation. Moreover, in the kidney proximal tubules, diabetes is induced aberrant DNA methylation [9, 10]. However, elevated glucose level is not the only reason for maladaptive epigenetic modifications in diabetes. Many other factors can modify epigenetic profiles, such as hypoxia, inflammation, and cytokines [11, 12]. The role of DNA methylation in DN has drawn more attention, partly because of the application of high-throughput sequencing technologies. By comparing type 2 diabetes patients with or without DN, several genes have clear differential methylation. The gene UNC13B is one of them, which has been suggested to mediate apoptosis in glomerular cells as a result of hyperglycaemia and could be relevant to the initiation and pathogenesis of DN [13]. Another team identified 187 gene targets that show differentially site-specific DNA methylation in DNA extracted from the saliva of patients with type 2 diabetes and end-stage kidney disease (ESRD) when compared to diabetic patients without ESRD [14]. In microdissected tubules obtained from patients with DN, DNA methylation profiles demonstrated differentially methylated genes implicated in fibrogenesis [15]. Another study analyzed DNA methylation in kidney tubular epithelial cells and showed significance differences of methylation in 1061 genes in DN patients compared to controls [16].

Involvement of DNA methylation in DN is also supported by experimental studies. It is reported that hypermethylation of RASAL1 increased Ras activation in fibroblasts, leading to proliferation and fibrosis [17]. Exposure of vascular endothelial cells to hyperglycemia led to changes in DNA methylation at several genes involved in endothelial cell dysfunction [18]. Taken together, these researches have identified the gene targets for DN and also prove the importance of DNA methylation in regulating fibrotic and other genes associated with DN.

## 3. Noncoding RNAs in DN

RNA used to be considered as an intermediate molecule in protein synthesis, which is no more than a template for protein synthesis. However, with the development of high-throughput platforms, the classical view of the molecular biology has changed [19, 20]. It has been reported that less than 2% of human genome is transcribed into RNA transcripts that can code protein [21–23]. It means that most of RNAs are noncoding RNAs (ncRNAs) separated into long ncRNAs (more than 200 nucleotides in length) and small ncRNAs (less than 200 nucleotides).

## 4. miRNA

MicroRNAs (miRNAs) are small noncoding RNA molecules capable of silencing mRNA targets. miRNAs contain 20~22 nucleotides and typically bind to the 3′ untranslated regions of target mRNA to promote translational repression and/or mRNA degradation [24]. It is reported that more than 2500 mature miRNAs are identified in humans and regulated at least 60% of protein-coding genes [25]. Hence, miRNAs can modulate the expression of numerous genes to alter key cellular functions and influence the course of various diseases. Some miRNAs are considered to have renal functions because they are enriched in kidney only [26]. Besides, miRNAs may have cell type and tissue-specific functions since different miRNA expression patterns were found in renal cortex and medulla [27].

Many miRNAs involved in DN have been identified [28]. Compared with nondiabetic control mice, several miRNAs (miR192, miR-200b/c, miR21, and miR-1207-5p) are upregulated in TGF-*β*1-treated murine mesangial cells and in renal glomeruli of mouse models of diabetes. The TGF-*β*1 pathway is a master regulator of renal fibrosis, which plays an important role in DN. Among these miRNAs, the best-studied one is miR-192, which has a conflicting expression in DN. miR-192 was reported to be upregulated in the glomeruli of streptozotocin- (STZ-) induced and db/db diabetic mice [29]. The upregulation of miR-192 expression is also shown in tubular cells treated with TGF-*β*1 [30]. However, other groups found a reduction in miR-192 in diabetic Apoe^−/−^ mice, which was associated with increased fibrosis [31, 32]. Investigation results explained that these complexities are due to cell type-specific effects of miRNA and differences in the animal models studied [33], while considering that decreasing of miR-192 is not pathogenic, because Kato et al. demonstrated that miR-192 knockout (KO) mice had no kidney problem and milder kidney injury under diabetes. Moreover, some studies had reported that the decreasing of miR-192 at later stage can be just a result of nonspecific degradation of RNAs in progression of DN but not pathogenic [34, 35]. It has reported that the level of miR-192 is increased in the early stage of DN, which can be prevented via suppressing this microRNA expression in the mice model of DN [36, 37]. Many articles describe the function of miR-192 in DN. First, miR-192 can lead to upregulation of Col1a2 and Col4a1 in mesangial cells. They are key genes associated with the pathogenesis of DN [29]. Second, miR-192 can also regulate other miRNAs, such as miR-216a/miR-217 and miR-200b/c. It is known that miR-200 family members can regulate Zeb1 and Zeb2. MiR-200 can regulate collagen expression and promote the autoregulation of TGF-*β*1 in murine mesangial cells by inhibiting Zeb1. Moreover, miR-216/miR-217 also related to TGF-*β*1-induced Akt activation and cellular hypertrophy, which are considered to be a pivotal feature of DN [38]. Third, miR-192 is reported to have an amplification loop with p53 in response to TGF-*β*1. In this study, the levels of TGF-*β*1, p53, and miR192 were all increased in the renal cortex of diabetic mice compared with control ones [34]. Generally, miR-192 is one of the major regulators in the pathologic mechanism of DN.

Similarly, miR-21 is known to modulate the expression and activities of important factors related to diabetic kidney disease. The mechanism of miR-21 regulating renal injury is targeting Smad7 because downregulation of miR-21 can restore Smad7 levels and suppressed activation of the TGF-*β* and NF-*κ*B signaling pathway [39]. In the renal cortex of OVE26 type 1 diabetic mice, miR-21 was upregulated and could target Pten and promote mTOR activation, which is related to DN.

## 5. lncRNA

Long noncoding RNAs are defined as a large and diverse group of non-protein-coding transcripts longer than 200 nucleotides [40]. Based on the association with nearby protein-coding genes, the lncRNAs can be separated into six groups: sense (overlapping a protein-coding gene), antisense (located in antisense orientation to a protein-coding gene), bidirectional promoter (transcribed within 1 kb of promoters antisense to the protein-coding transcript), intronic (transcribed from an intron of a protein-coding gene), intergenic (between two protein-coding transcripts), and enhancer (transcribed from an enhancer region of a protein-coding gene) [41]. Circular RNAs also have been identified as they form covalently enclosed circular structure, which usually come from splicing of a protein-coding gene [42]. According to the mechanism of lncRNAs, they can be classified into four categories—signal, decoy, guide, and scaffold [43]. Accumulating evidence has demonstrated that the noncoding RNA (ncRNA) affects transcription, pre-mRNA processing, and translation [43, 44].

Unlike microRNA, there are only a few reports that have shown the relationship of lncRNAs with DN. First, as a host gene, ncRNA RP23 together with miR-216 and miR-217 were induced by TGF-*β*1 [45]. Another host gene ncRNA CJ241444 can be coregulated with miR-192 and is induced by TGF-*β*1. This mechanism involved the Smad transcription factors, protein C-ets-1, and histone acetylation [46]. Gene set enrichment analyses (GSEA) show that a megacluster of nearly 40 microRNAs and their host long noncoding RNA transcript (lnc-MGC) are coordinately increased in the glomeruli of mouse models of DN. Inhibition of the host lncRNA decreased the expression of the cluster miRNAs and attenuated early features of DN in vitro in mesangial cells and in vivo in mice [47]. Second, another study reported that 21 common lncRNAs were upregulated in wild-type, but downregulated in Smad3 knockout, kidneys in both disease models in which progressive renal inflammation and fibrosis were abolished when the Smad3 gene was deleted or suppressed [48]. An unbiased RNA-sequencing (RNA-seq) analysis of kidney glomeruli identified that lncRNA Tug1 are considered as a differentially expressed lncRNA in the diabetic milieu. This Tug1 gene is shown to regulate mitochondrial bioenergetics in diabetic nephropathy [49]. Third, one lncRNA named Malat1 may have relationship with DN. It was first identified as a prognostic marker of survival in early-stage non-small-cell lung cancers. However, the expression of Malat1 is not restricted to cancer cell, and it is ubiquitously expressed in many normal cells and tissues [50]. Diabetes is usually recognized as the vascular disease characterized by vasoregulation change, increased generation of reactive oxygen intermediates, inflammatory activation, and altered barrier function [51]. It has been reported that the MALAT1 level is significantly upregulated in diabetic animal models. Moreover, MALAT1 expression is upregulated in the kidneys of diabetic mice. The mechanism of the result is lncRNA MALAT1 inhibition attenuates the inflammatory response in condition of hyperglycaemia via serum amyloid antigen3 (SAA3) [52]. Generally, the relationship of lncRNA and DN seems an exciting emerging field that is expected to have more findings.

## 6. Histone Modifications in DN

Histone posttranslational modifications (PTMs) regulate gene expression chromatin structure. Important PTMs of histone include lysine acetylation (Kac), lysine methylation (Kme), and phosphorylation. They are mostly in the exposed amino-terminal tails [53]. Histone Kac is catalyzed by histone acetyltransferases such as p300 and CBP, which also act as transcription coactivators. Lysine is deacetylated by HDACs. Kme is carried out by lysine methyl transferases and demethylation by lysine demethylases [54].

Recently, some papers have shown the role of histone modifications in diabetes and diabetic complications, including DN. Histone PTMs are among the best-characterized epigenetic modifications with respect to diabetes and are clearly implicated in the reduction in the expression of genes implicated in DN [55]. The role of histone PTMs in DN has been investigated in vitro and in vivo. The functional role of histone H3 lysine methylation (H3Kme) in TGF-*β*1-mediated extracellular matrix (ECM) gene expression in mesangial cells has been shown under normal and high-glucose (HG) conditions [56]. Another study reported that high glucose inhibited DNA methylation and increased H3K9ac at the promoter of the redox regulating protein p66 and upregulated p66shc expression in diabetic murine kidney [57]. Histone modifications also showed connection with microRNA. Histone acetylation by p300 activated by Akt is involved in induction of miR-192 in diabetic nephropathy. These findings provide insight into the regulation of miRNAs through signaling-mediated changes in epigenetic histone acetylation under normal and disease states [46].

## 7. Epigenetic and ncRNA-Based Therapy

As the leading cause of renal failure, DN requires renal replacement therapy worldwide. However, effective methods to identify and halt progression of pathophysiological changes of DN remain elusive [58, 59]. In the past, proteinuria, renal pathology, and renal function are major diagnosis for DN. Recently, with the development of new technologies, such as quantitative real-time PCR, microarray, and high-throughput sequencing, it is possible for us to use ncRNA as the biomarker for DN. NcRNAs have their own characteristics. Firstly, miRNAs have consistency in particular disease and have tissue specificity. Secondly, miRNAs are highly expressed in urine and have stability storage in tissue [60, 61]. Thirdly, in some cases, miRNA changes expression level in the early stage of DN, which related to fibrosis [62]. For example, Mohan et al. reported that miR-451-5p and miR-16 in diabetes appeared to be protective against diabetes-induced kidney fibrosis, while UE miR-451-5p may hold prognostic value as an early and sensitive noninvasive indicator of renal disease [63]. Similar to miRNA, lncRNA also can help in serial monitoring of diabetic patients. On the other hand, there are some disadvantages of using ncRNA as biomarkers to diagnose DN. With developing of renal failure, low miRNA level may be due to decreasing of synthesis capacity. So it cannot be attributed to downregulated expression.

The current standard therapy of diabetic nephropathy involves intensive treatment of hyperglycemia and strict blood pressure control, mainly via blockade of the renin-angiotensin system (RAS) [64]. As discussed earlier in this review, epigenetic regulation of DNA and histones plays important parts in DN. It has been reported that histone methylation has connection with metabolic memory. Preliminary work in endothelial cells has shown that transient episodes of hyperglycemia can induce changes in gene expression that are dependent on histone modifications and that these changes persist after return to normoglycemia [65]. Attention has been drawn to additional beneficial effects of epigenetic modifications. In connection with abnormal expression of miRNA in DN, targeting regulation of miRNA expression is regarded as a novel therapy for DN treatment.

## 8. Conclusions

The pathogenesis of DN involves complicated interactions between metabolic and hemodynamic factors. Increasing evidence suggests a critical role for epigenetic modifications in DN. In this review, we have highlighted some emerging mechanisms including DNA methylation, noncoding RNA, and histone modification. Rapid developments of high-throughput genome-wide screening techniques have greatly broadened the understanding of genetic and epigenetic changes in DN. Unlike genetic changes, epigenetic changes are reversible, which means it gives an opportunity for therapeutic development. However, the mechanism of action of these inhibitors is not fully clear, and more work is needed before understanding it. Epigenetic therapy has been expected to obtain more achievements in the future. Further work in this field of research has a chance to make a distinguished influence on the therapy of DN.
